# Supplementary material for: Krill Oil’s Protective Benefits against Ultraviolet B-Induced Skin Photoaging in Hairless Mice and In Vitro Experiments
Source: Mar Drugs. 2023 Aug 30;21(9):479. doi: 10.3390/md21090479 (PMC10533088; doi:10.3390/md21090479)
Supplement: Supplementary file 1 [file marinedrugs-21-00479-s001.zip › marinedrugs-2572838-supplementary.pdf]

Figure S1: GC analysis of KO for EPA+DHA contents

[Krill oil; EPA+DHA]

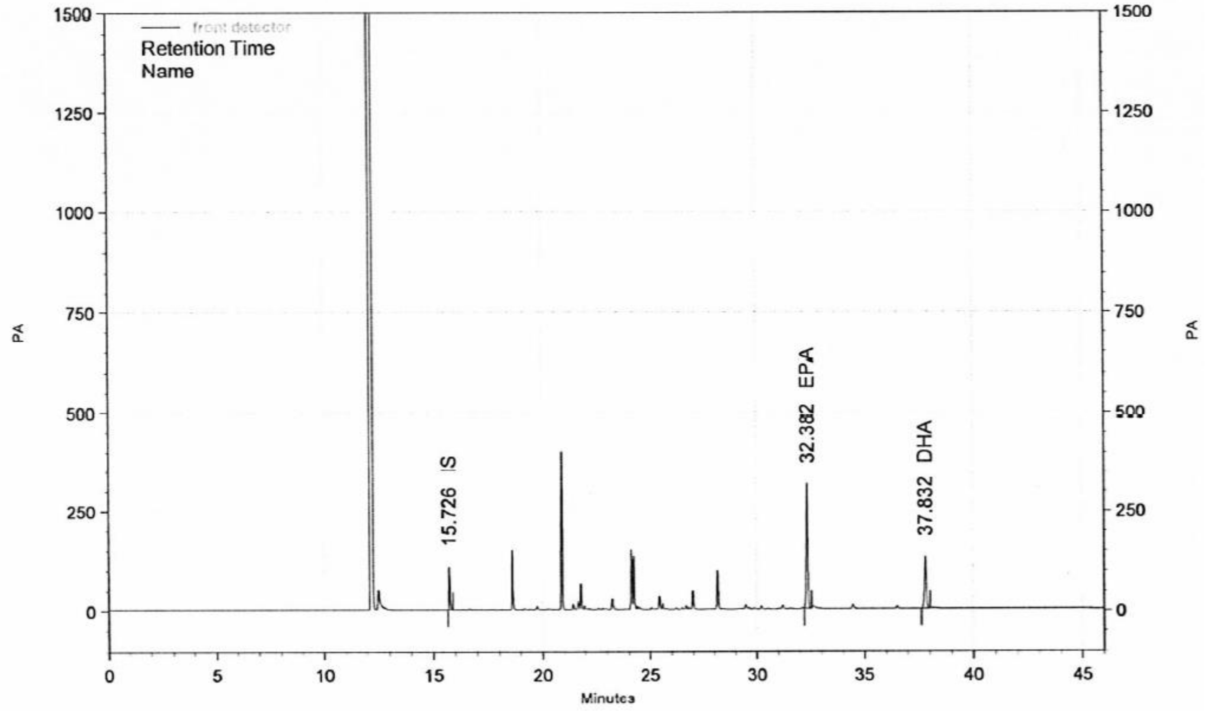

[Blank; EPA+DHA]

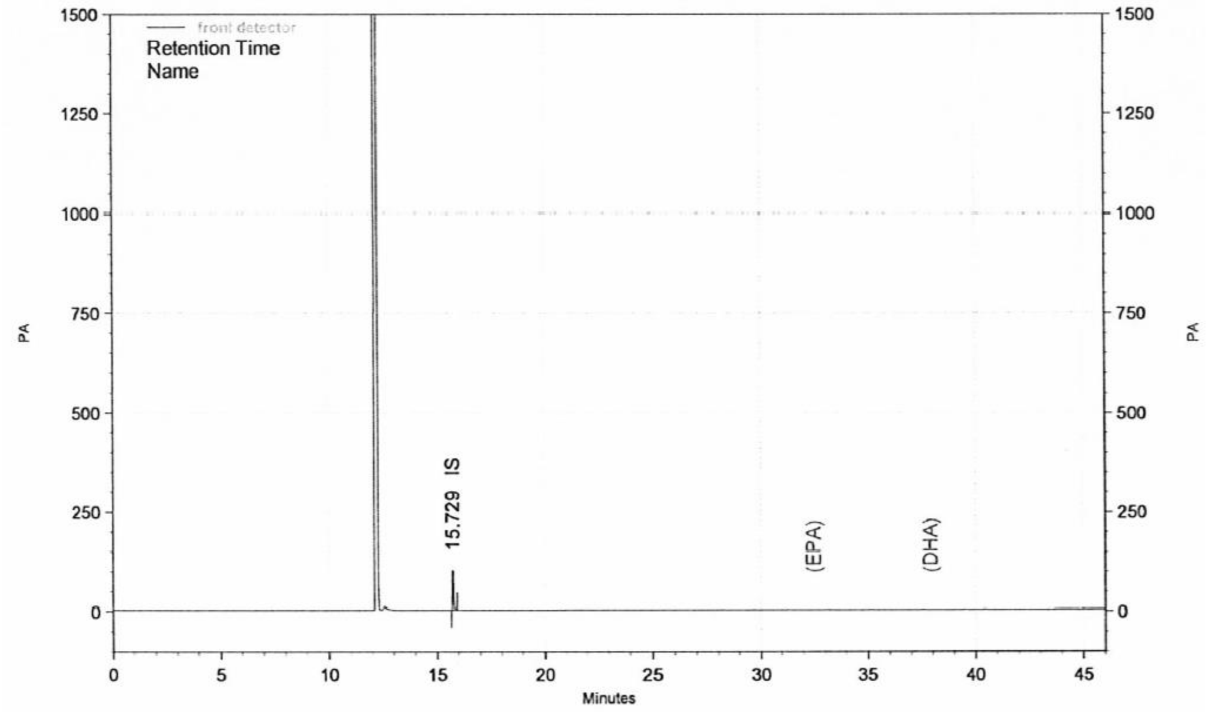

[Standard; EPA+DHA]

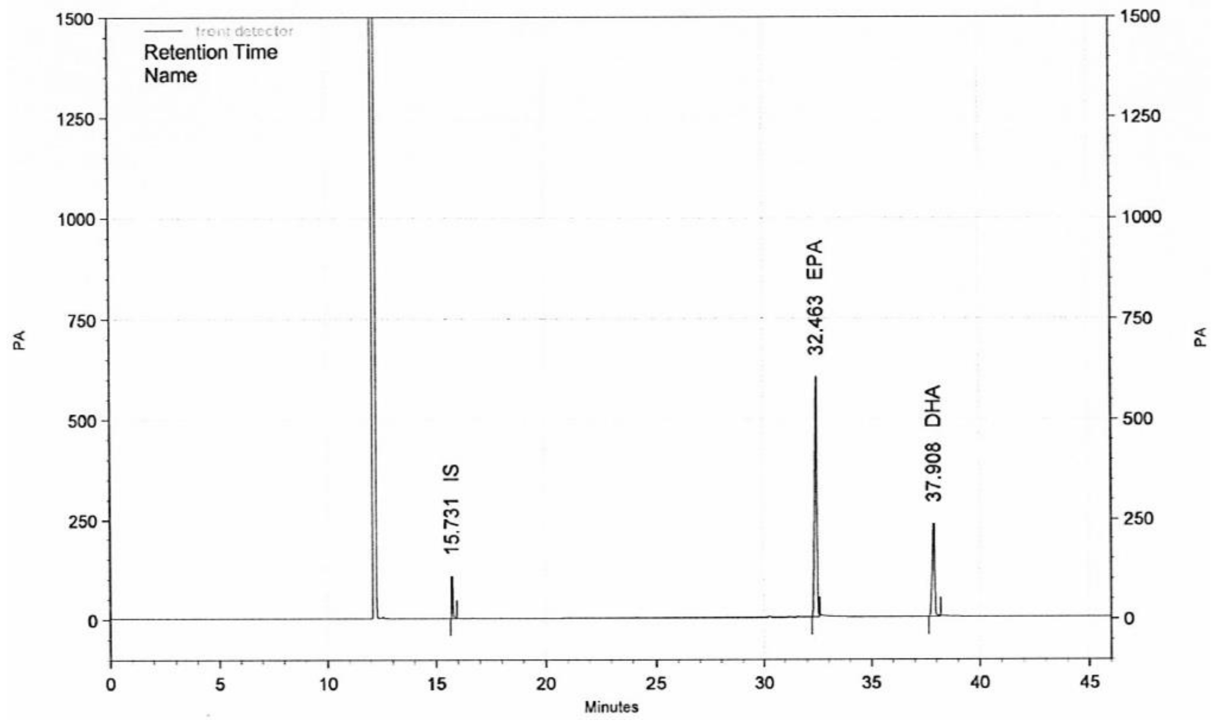

Table S1. Primer sequences for real-time RT-PCR

Target	5' – 3'	Sequence
Hyaluronic acid synthase 1	Sense	GCATGGGCTATGCTACCAAGTAT
	Antisense	AGGAGGGCGTCTCCGAGTA
Hyaluronic acid synthase 2	Sense	GACCCTATGGTTGGAGGTGTTG
	Antisense	ACGCTGCTGAGGAAGGAGATC
Hyaluronic acid synthase 3	Sense	AGACCGAGCTAGCCTTCCTAGT
	Antisense	TAATGGCCAGATACAGCATGAG
Collagen type I alpha 1	Sense	GCGGTAACGATGGTGCTGTT
	Antisense	CTTCACCCTTAGCACCAAC
Collagen type I alpha 2	Sense	ATTGTCGCCAGTGAG
	Antisense	CTGGTCCTGCTGGT
TGF-β1	Sense	GCAACATGTGGAACTCTACCAGAA
	Antisense	GACGTCAAAAGACAGCCACTCA
p38 MAPK	Sense	CGTTGTTTCCTGGTACAGACC
	Antisense	CCATTTCTTCTTGGTCAAGGG
Protein kinase B (AKT)	Sense	TACTCATTCCAGACCCACGA
	Antisense	GAGGTTCTCCAGCTTCAGGT
Matrix metalloprotease-1	Sense	AAGGTTAGCTTACTGTCACACGCTT
	Antisense	CGACTCTAGAAACACAAGAGCAAGA
Matrix metalloprotease-9	Sense	CCCGGACCAAGGATACAG
	Antisense	GGCTTTCTCTCGGTACTG
Matrix metalloprotease-13	Sense	CATCCATCCCGTGACCTTAT
	Antisense	GCATGACTCTCACAATGCGA
Glutathione reductase	Sense	TGCGTGAATGTTGGATGTGTACCC
	Antisense	CCGGCATTCTCCAGTTCCTCG
Nox2	Sense	AGCTATGAGGTGGTGATGTTAGTGG
	Antisense	CACAATATTTGTACCAGACAGACTTGAG
β-actin	Sense	AGCTGCGTTTTACACCCTTT
	Antisense	AAGCCATGCCAATGTTGTCT

RT-PCR = reverse transcription polymerase chain reaction; TGF-β1= transforming growth factor (TGF)-β1; MAPK = Mitogen-activated protein kinase; Nox2 = *gp91phox* subunit of the phagocyte NADPH oxidase
